# Comparative evaluation of *Populus* variants total sugar release and structural features following pretreatment and digestion by two distinct biological systems

**DOI:** 10.1186/s13068-017-0975-x

**Published:** 2017-11-30

**Authors:** Vanessa A. Thomas, Ninad Kothari, Samarthya Bhagia, Hannah Akinosho, Mi Li, Yunqiao Pu, Chang Geun Yoo, Sivakumar Pattathil, Michael G. Hahn, Arthur J. Raguaskas, Charles E. Wyman, Rajeev Kumar

**Affiliations:** 1 Department of Chemical and Environmental Engineering, Bourns College of Engineering, University of California Riverside, Riverside, CA 92507 USA; 2 Center for Environmental Research and Technology (CE-CERT), Bourns College of Engineering, University of California Riverside, Riverside, CA 92507 USA; 3 School of Chemistry and Biochemistry & Renewable Bioproducts Institute, Atlanta, GA 30332 USA; 4 BioEnergy Science Center, Oak Ridge National Laboratory, Oak Ridge, TN 37831 USA; 5 UT-ORNL Joint Institute for Biological Sciences, Oak Ridge National Laboratory, Oak Ridge, TN 37831 USA; 6 Complex Carbohydrate Research Center, University of Georgia, Athens, GA 30602 USA; 7 Department of Chemical and Biomolecular Engineering, Center for Renewable Carbon, University of Tennessee, Knoxville, TN 37996 USA; 8 Department of Forestry, Wildlife, and Fisheries, Center for Renewable Carbon, University of Tennessee, Knoxville, TN 37996 USA; 9 Present Address: Mascoma LLC (Lallemand Inc.), 67 Etna Road, Lebanon, NH 03766 USA

**Keywords:** *Populus*, Natural variants, Hydrothermal pretreatment, Structural changes, Enzymatic hydrolysis, Consolidated bioprocessing, *Clostridium thermocellum*

## Abstract

**Background:**

*Populus* natural variants have been shown to realize a broad range of sugar yields during saccharification, however, the structural features responsible for higher sugar release from natural variants are not clear. In addition, the sugar release patterns resulting from digestion with two distinct biological systems, fungal enzymes and *Clostridium thermocellum*, have yet to be evaluated and compared. This study evaluates the effect of structural features of three natural variant *Populus* lines, which includes the line BESC standard, with respect to the overall process of sugar release for two different biological systems.

**Results:**

*Populus* natural variants, SKWE 24-2 and BESC 876, showed higher sugar release from hydrothermal pretreatment combined with either enzymatic hydrolysis or *Clostridium thermocellum* fermentation compared to the *Populus* natural variant, BESC standard. However, *C. thermocellum* outperformed the fungal cellulases yielding 96.0, 95.5, and 85.9% glucan plus xylan release from SKWE 24-2, BESC 876, and BESC standard, respectively. Among the feedstock properties evaluated, cellulose accessibility and glycome profiling provided insights into factors that govern differences in sugar release between the low recalcitrant lines and the BESC standard line. However, because this distinction was more apparent in the solids after pretreatment than in the untreated biomass, pretreatment was necessary to differentiate recalcitrance among *Populus* lines. Glycome profiling analysis showed that SKWE 24-2 contained the most loosely bound cell wall glycans, followed by BESC 876, and BESC standard. Additionally, lower molecular weight lignin may be favorable for effective hydrolysis, since *C. thermocellum* reduced lignin molecular weight more than fungal enzymes across all *Populus* lines.

**Conclusions:**

Low recalcitrant *Populus* natural variants, SKWE 24-2 and BESC 876, showed higher sugar yields than BESC standard when hydrothermal pretreatment was combined with biological digestion. However, *C. thermocellum* was determined to be a more robust and effective biological catalyst than a commercial fungal cellulase cocktail. As anticipated, recalcitrance was not readily predicted through analytical methods that determined structural properties alone. However, combining structural analysis with pretreatment enabled the identification of attributes that govern recalcitrance, namely cellulose accessibility, xylan content in the pretreated solids, and non-cellulosic glycan extractability.

**Electronic supplementary material:**

The online version of this article (10.1186/s13068-017-0975-x) contains supplementary material, which is available to authorized users.

## Background

Carbon–neutral fuels and chemicals sustainably derived from lignocellulosic biomass can play a significant role in mitigating the challenges associated with climate change and meeting the planet’s energy and material demands [1, 2]. However, capital- and energy-intensive pretreatment and preprocessing steps together with high dosages of costly fungal enzymes are required to overcome the native resistance of biomass to deconstruction (“recalcitrance”) and realize commercially relevant yields. Although several pretreatment methods can render biomass digestible for its conversion to sugars, hydrothermal pretreatment using just liquid hot water as a catalyst is one of the most attractive options due to its operational simplicity and acceptable performance for a variety of feedstocks [3, 4]. Nonetheless, because the high loadings of expensive enzymes needed to deconstruct hydrothermally pretreated solids offset their advantages [2], it is desirable to develop processes that require minimal (or no) external fungal enzymes. Consolidated bioprocessing (CBP) using the thermophilic anaerobic bacterium *Clostridium thermocellum* [5, 6] can potentially result in significant cost savings by combining enzyme production, saccharification, and fermentation in one pot.

Although genetic manipulation provides another option to reduce biomass recalcitrance to biological conversion [7, 8], it has also been observed that naturally occurring variants in plants differ in digestibility from their control counterparts [9, 10]. In the past, *Populus* natural variant lines have been shown to yield higher sugar release than the standard lines [10]. However, the structural features in these natural variants that are responsible for the observed increases in sugar release are unclear [11]. In addition, the sugar release patterns for such lines applying two distinct biological systems, fungal enzymes and *C. thermocellum*, have not been evaluated and compared yet.

In this study, the effects of plant lines and crop variance on carbohydrate sugar release by two distinctive biological systems were investigated to determine how crop variance influenced final conversion and identify possible attributes that made one plant more easily deconstructed than the other. To evaluate the influence of feedstock diversity within the *Populus* genus, deconstruction and key features of two natural variants that displayed reduced recalcitrance, SKWE 24-2 and BESC 876, were compared to those of the highly recalcitrant BESC standard line. Both SKWE 24-2 and BESC 876 carry naturally occurring mutations in the 5-enolpyruvylshikimate-3-phosphate (EPSP) synthase gene that lead to the conversion of the synthase into a transcriptional regulator for many enzymes involved in lignin biosynthesis and amino acid production [12–14]. These low recalcitrant *Populus* lines were chosen based on prior work by Bhagia et al. [12, 13], who determined the recalcitrance of 22 natural variant *Populus* lines as measured by total sugar release after pretreatment and enzymatic hydrolysis of the biomass isolated from these lines. In the previous study (and in the work presented here), Stage 1 and Stage 2 refer to pretreatment and biological digestion, respectively. Hydrothermal pretreatment was applied to each variant at a severity factor, i.e., combination of pretreatment temperature and time [15], of 3.6 at the temperatures of 140, 160, and 180 °C to determine the effect of pretreatment temperature on sugar yield trends across the *Populus* lines. Sugar yields were measured in terms of total glucan plus xylan yields after 24 h of enzymatic hydrolysis using 75 mg protein of cellulase and 25 mg protein of xylanase. Based on the results of the previous studies, BESC 876 and SKWE 24-2 were chosen to represent a low recalcitrant variant from paralogs 1 and 2, respectively, of the *EPSP* gene for comparison with BESC standard *Populus*, which was identified to be the most recalcitrant natural variant of the 22 lines tested. Thus, these three lines provided biomass showing low and high recalcitrance.

The three *Populus* lines, BESC standard, SKWE 24-2, and BESC 876, were hydrothermally pretreated at 200 °C for 22.7 min. These conditions were previously found to be optimal for achieving maximum sugar release from the BESC standard line after subsequent hydrolysis by both *C. thermocellum* and fungal enzymes at 65 mg protein/g glucan of pretreated biomass [16]. Following pretreatment, each line was incubated with a commercial preparation of fungal cellulases, Accellerase^®^1500, and a wild-type CBP organism, *C. thermocellum*, for 7 days at their respective optimal incubation conditions to determine maximum sugar release. Moderate and high enzyme loadings of 15 and 65 mg protein/g glucan, respectively, were applied to the pretreated biomass.

Biomass material composition, pretreatment solid yield, pretreatment material balances, and sugar release by each digestion regime were determined. Additionally, ultrastructural features of the raw, pretreated, and biological residual *Populus* were determined to identify plant cell wall structures and chemistry that hindered the attainment of complete release of glucose and other cell wall sugars. The following properties were evaluated: cellulose crystallinity, cellulose accessibility, non-cellulosic glycan epitope content and extractability, cellulose, hemicellulose, and lignin degree of polymerization/molecular weight, and relative abundance of lignin subunits. To catalog how these properties changed throughout processing, the untreated, hydrothermally pretreated, and residual *Populus* solids after digestions were characterized. Cell wall properties were evaluated in light of the sugar release results to see if the structural attributes that correlated with the reduced recalcitrance could be identified which could be used for easier selection of favorable plant lines and to potentially provide insight into the mechanistic workings of enzymes and *C. thermocellum* by contrasting the properties of their residues.

## Results and discussion

### Compositional analysis and overview of pretreatment of *Populus* natural variants

The compositions and cellulose crystallinity for raw, untreated, and hydrothermally pretreated solids are summarized in Table 1. These analyses were performed to establish a baseline for comparing the *Populus* lines with respect to cell wall composition and cellulose structure prior to digestion with either enzymes or *C. thermocellum*. While BESC standard had a slightly higher glucan content than SKWE 24-2 or BESC 876 when evaluating the raw *Populus* biomass compositions, statistical analysis showed that glucan, xylan, and lignin contents did not vary significantly between the lines (whether raw or pretreated) with the exception of the xylan content of the pretreated *Populus* which was found to be statistically different between the three natural variants (Student’s *t* test, *p* < 0.05). Although cellulose crystallinity of unpretreated lines were significantly different (Additional file 1: Table S1), no statistical difference was seen after pretreatment. As a result, raw material composition and cellulose crystallinity after pretreatment were not considered to be the indicators of reduced recalcitrance. The statistically significant lower xylan content after pretreatment for SKWE 24-2 and BESC 876 was noted as a potential marker of reduced recalcitrance.

**Table 1:** *Populus* solid composition and cellulose crystallinity

*Populus* type	Composition (%)			Cellulose crystallinity (%)
	Glucan (± 1.0)	Xylan (± 0.5)	Lignin (± 1.0)	
Raw				
BESC standard	48.0	17.4	22.1	55.3
BESC 876	42.8	16.6	23.5	54.5
SKWE 24-2	44.3	17.4	22.4	51.6
Hydrothermally pretreated (200 °C and 22.7 min)				
BESC standard	66.8	4.2	28.2	62.3
BESC 876	67.0	3.1	29.8	63.0
SKWE 24-2	67.1	3.1	28.1	60.8

**Fig. 1:**
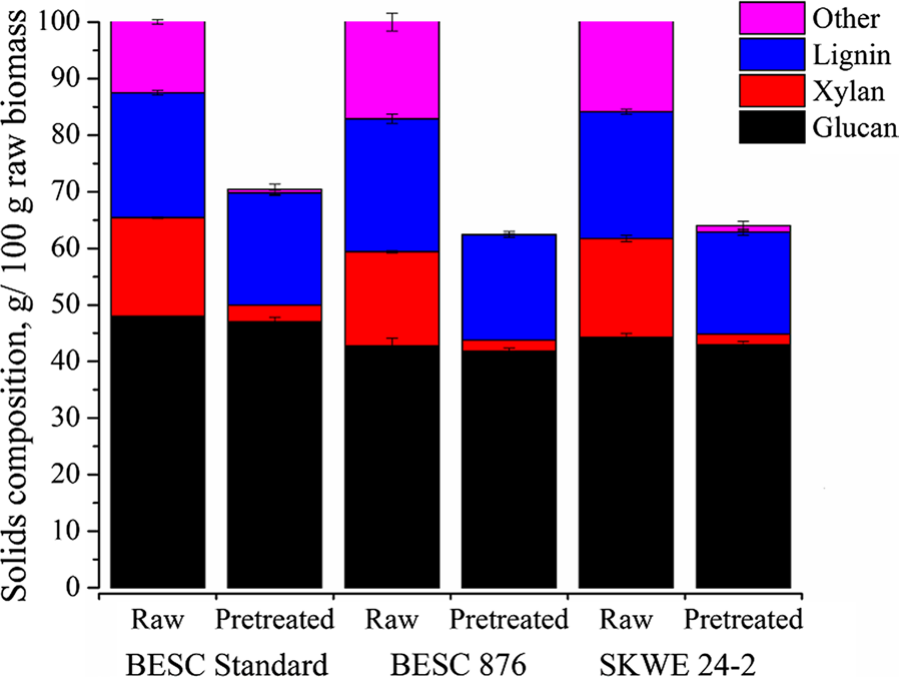
Tracking the masses of glucan, xylan, Klason lignin, and other compounds in raw and pretreated biomass from *Populus* natural variants on a basis of 100 g of each variant

The contents of glucan, xylan, Klason lignin, and other compounds (such as ash, extractives, acetyl content, and nitrogen) based on 100 g of raw *Populus* and solid yield are shown in Fig. 1. Because the values are normalized, the percent total solid yield in terms of grams of solids in the pretreated biomass per 100 g of raw biomass for the pretreated *Populus* lines is equal to the total height of each stacked bar. These results show that the BESC 876 and SKWE 24-2 lines had slightly lower total solid yields than BESC standard after hydrothermal pretreatment at 200 °C for 22.7 min. This was partly due to the fact that these lines initially had slightly lower, though not statistically significant, amounts of glucan than the BESC standard line.

**Fig. 2:**
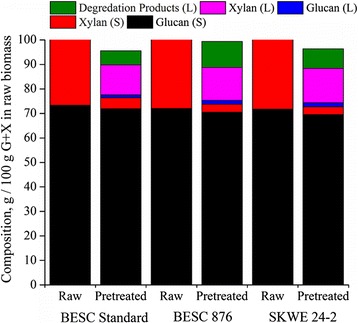
Stage 1 glucan (G) and xylan (X) material balances based on 100 g of glucan plus xylan in each raw *Populus* variant for liquid (L) and solid (S) streams produced by hydrothermal pretreatment of *Populus* natural variants at 200 °C for 22.7 min. The sugar degradation products measured in the pretreatment liquor included levulinic acid, formic acid, 5-HMF, and furfural and were adjusted to the corresponding amount of glucan or xylan based on the appropriate stoichiometry. Note: Formic acid has the potential to form from either glucose or xylose; however, it was assumed to form from xylose since xylan/xyloglucan degradation was predominant while glucan degradation was minimal

Figure 2 shows the distribution of glucan, xylan, and their degradation products between the liquid and solid fractions from hydrothermal pretreatment on the basis of 100 g of glucan plus xylan in the respective untreated *Populus* variants. Figure 2 shows that the composition on a basis of 100 g glucan plus xylan (i.e., the sugar composition) is quite similar between the solids of all three lines before and after pretreatment, establishing a consistent compositional baseline between the *Populus* lines for evaluating Stage 2 digestibility. Figure 2 also shows the glucan and xylan material balances around pretreatment, revealing that hydrothermal pretreatment of BESC 876 and SKWE 24-2 degraded more sugar to 5-HMF, furfural, levulinic acid, and formic acid than BESC standard. Despite more sugar degradation products being measurable in the pretreatment liquor for the two low recalcitrant natural variants, the three *Populus* lines gave approximately the same recovery of glucan plus xylan monomers and oligomers for the combined solid and liquid streams from pretreatment (see Table 2). This observation suggests that there were likely additional degradation products that could not be quantified by the assays employed to generate the data in Fig. 2, resulting in lower net xylan mass balance closures while maintaining equivalent recoveries as demonstrated in Table 2. Although the xylan material balances added up to less than 100%, their effect on the overall glucan plus xylan material balance was less than that of the glucan balance alone, since xylan made up only a quarter of the initial sugar content compared to glucan which made up the remaining three quarters. The xylan material balance closure was greatest for BESC 876, followed by SKWE 24-2 and BESC standard. Glucan plus xylan material balances were close to 100 ± 5% for all *Populus* lines, which is within reasonable error, and glucan plus xylan recovery (monomers and oligomers) was approximately 90% of that originally present for all *Populus* lines.

**Table 2:** Stage 1 glucan and xylan material balances and recoveries

*Populus* type	Material balance closure^a^ (%)			Recovery^b^ (%)		
	Glucan	Xylan	Glucan + Xylan	Glucan	Xylan	Glucan + Xylan
Hydrothermally pretreated						
BESC standard	100	83	96	100	63	90
BESC 876	101	95	99	100	60	89
SKWE 24-2	100	86	96	99	61	88

^a^Includes glucan, xylan, and their degradation products in pretreatment liquor and pretreated solids

^b^Includes glucan and xylan only in pretreatment liquor and pretreated solids

### Biological digestion of *Populus* natural variants

**Fig. 3:**
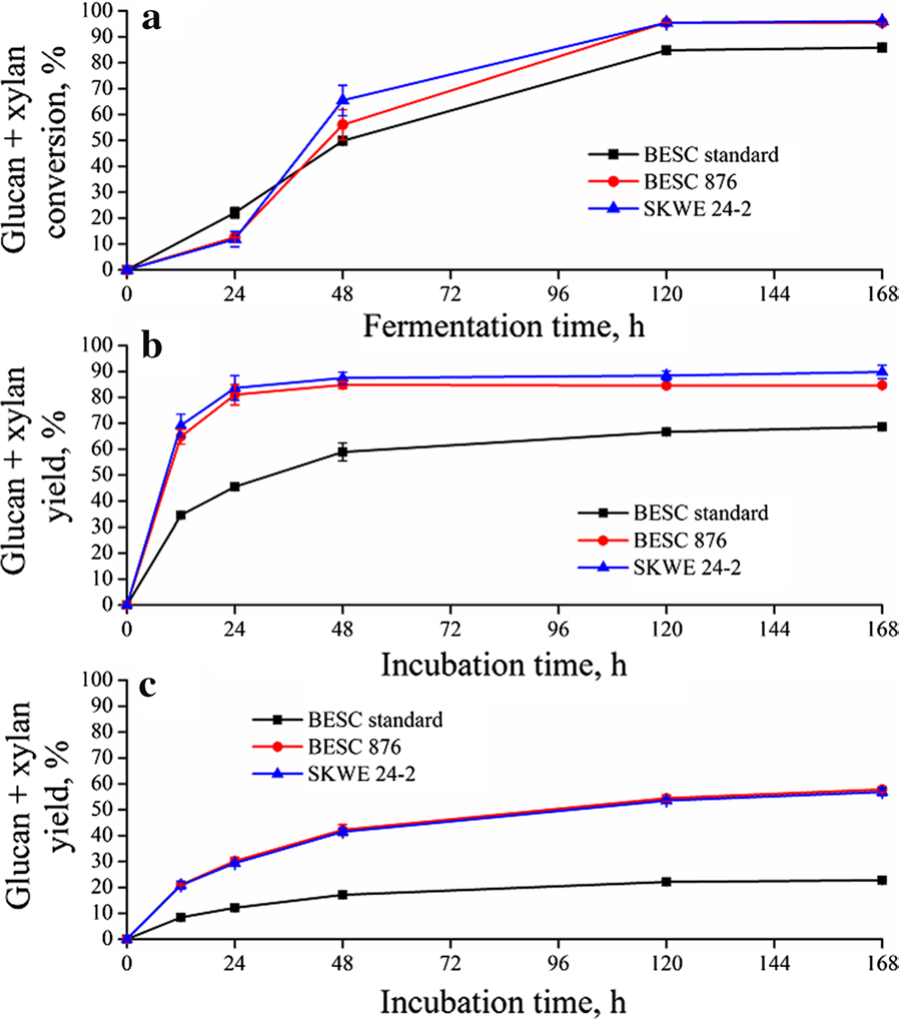
**a** Stage 2 *C. thermocellum* CBP glucan plus xylan conversions and enzymatic hydrolysis glucan plus xylan yields for a cellulase loading of (**b)** 65 mg protein/g glucan in the pretreated biomass (**c**) and 15 mg protein/g glucan in the pretreated biomass as a function of time for *Populus* natural variants hydrothermally pretreated at a severity factor of 4.3 (200 °C and 22.7 min). Note: Protein loadings were based on glucan content in the pretreated biomass. Fermentations were sampled at 24, 48, 120, and 168 h and enzymatic hydrolysis reactions were sampled at 12, 24, 48, 120, and 168 h

To determine the recalcitrance of pretreated variants to deconstruction by fungal enzymes versus *C. thermocellum* CBP, the solids were washed and fed to Stage 2, biological digestion. The 168-h Stage 2 enzymatic hydrolysis yields and the *C. thermocellum* conversion profiles are shown in Fig. 3 for the three hydrothermally pretreated *Populus* lines. *C. thermocellum* achieved the highest sugar release from the pretreated solids for all variants given sufficient time. Specifically, *C. thermocellum* removed 95% of the available Stage 2 glucan and xylan after 120 h for SKWE 24-2 and BESC 876, compared to approximately 86% for BESC standard. By comparison, the higher enzyme dose of 65 mg of protein/g glucan in pretreated biomass was capable of producing only 90, 85, and 69% yield, respectively, for the three *Populus* lines. While *C. thermocellum* was able to release the greatest amount of sugar, faster initial hydrolysis rates were achieved by utilizing such a large enzyme dose. However, this advantage was lost when enzyme loadings were reduced to 15 mg of protein/g glucan in the pretreated biomass. It is important to recognize that these enzyme loadings would cost approximately $3.00 and $0.75/gal of ethanol produced [17], respectively, far more than can be justified for a process catalyst.

Although BESC standard appeared to be initially more digestible by the CBP system, BESC 876 and SKWE 24-2 realized higher final sugar release than BESC standard for both enzyme loadings and CBP, proving that these variants were indeed lower recalcitrant lines. Sugar release from SKWE 24-2 and BESC 876 were similar for the fungal enzyme and organismal digestion systems (Fig. 3a, b), while BESC standard had significantly lower glucan plus xylan yields for a fungal enzyme loading of 65 mg protein/g glucan in the pretreated biomass compared to *C. thermocellum*. At the lower enzyme loading of 15 mg protein/g glucan, only 23% of glucan and xylan in BESC standard was converted to sugars compared to more than 50% from the low recalcitrant variants after 168 h.

**Fig. 4:**
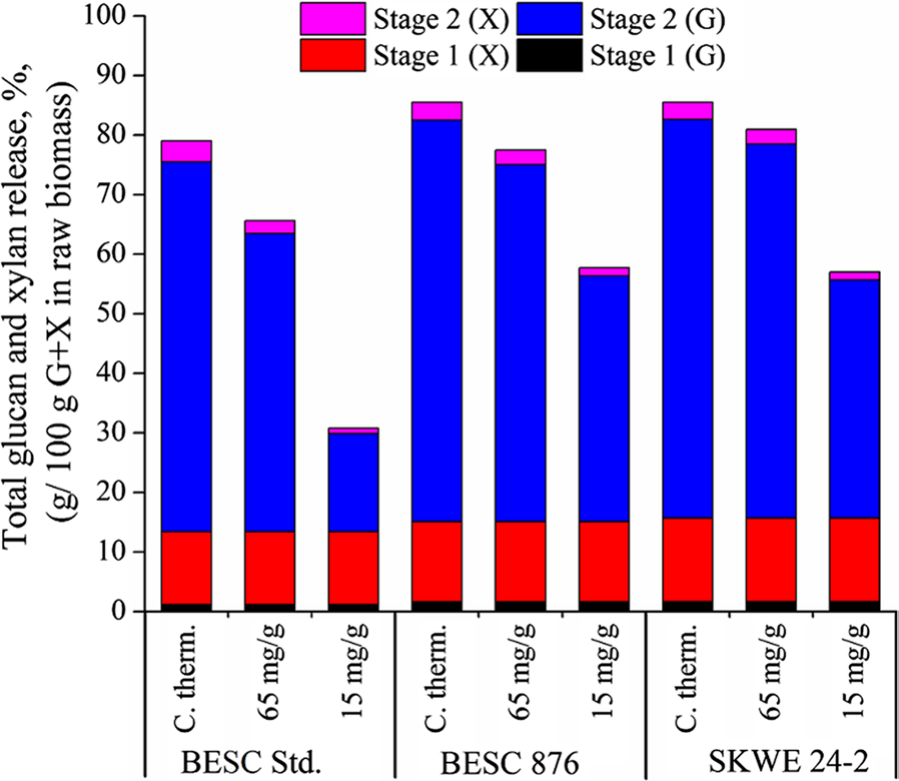
Amounts of glucan (G) and xylan (X) released from solids during pretreatment (Stage 1) and biological digestion (Stage 2) for fungal enzymes and *C. thermocellum* after 168 h. Sugar release was normalized to 100 g of glucan plus xylan in the raw biomass for each respective *Populus* variety

To determine overall process performance, Stage 1 plus Stage 2 glucan and xylan release was determined for each *Populus* line and biological catalyst after 168 h reaction time. These results are presented in Fig. 4. Again, *C. thermocellum* was the most effective at digesting the *Populus* lines with an almost 85% overall glucan plus xylan release for SKWE 24-2 and BESC 876 compared to 80% for BESC standard. These results were again normalized to grams per 100 g of glucan and xylan in the raw biomass to highlight the goal of conservation and conversion of all raw sugars, primarily those from glucan and xylan. Our results demonstrate that applying plant genetics to rationally select feedstocks with naturally reduced recalcitrance can improve overall sugar release. In the specific case here, selection of appropriate crop lines resulted in a 5% improvement in overall sugar release for the process. It may be possible to further increase this gain in total sugar release from the two low recalcitrant lines by reducing the pretreatment temperature while maintaining a constant severity (thus, increasing pretreatment time), as this method has been successful in conserving Stage 1 xylan in *Populus* [16].

### Structural and chemical characterization of raw and pretreated *Populus* solids and biological residues

A suite of characterization methods was applied to the raw, pretreated, and biological residues in search of changes in *Populus* features capable of relating recalcitrance to feedstock, pretreatment, or biological catalyst.

## Glycan extractability and their impact on recalcitrance

**Fig. 5:**
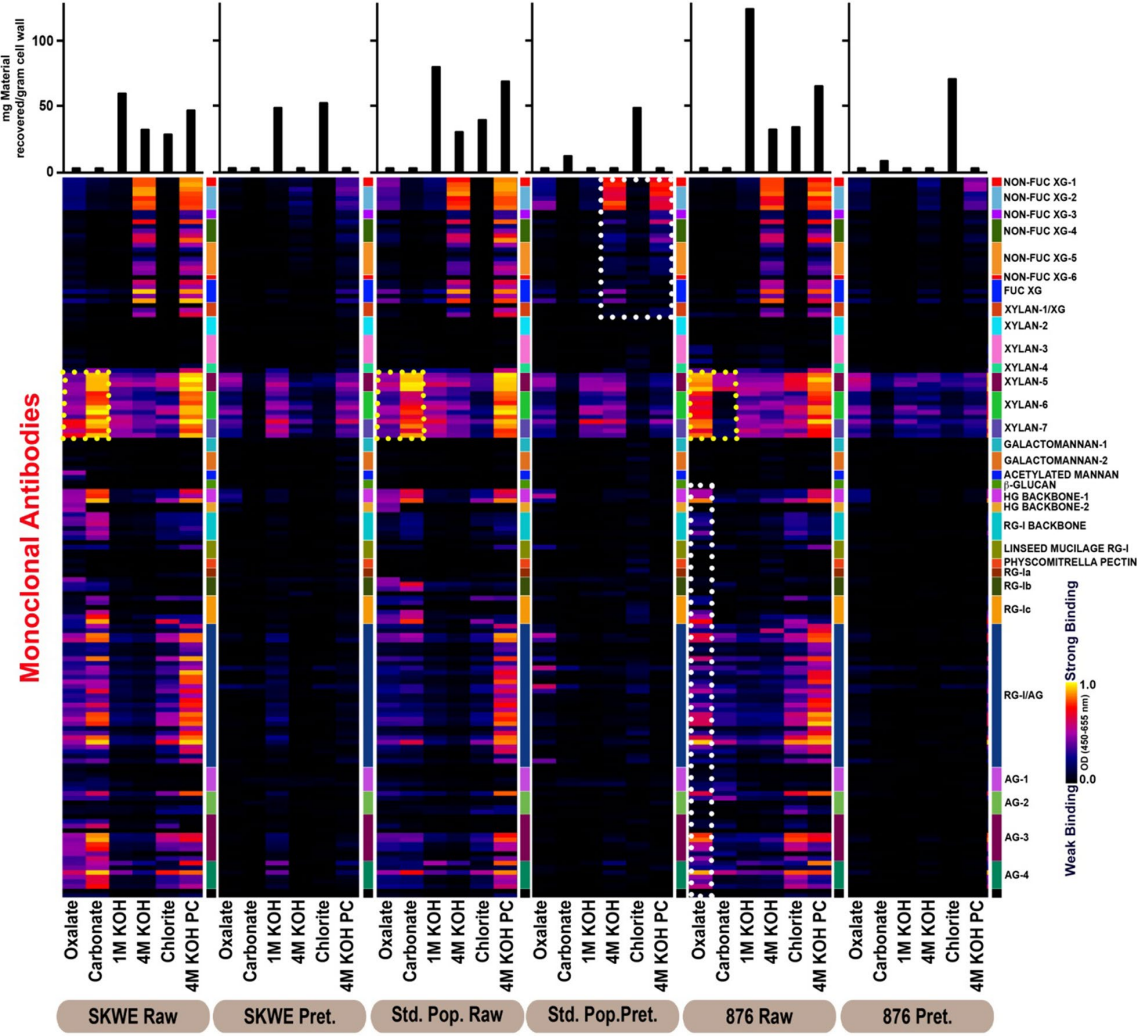
Glycome profiles for solids from raw and hydrothermally pretreated *Populus* natural variant lines resulting from subjecting the samples to sequential chemical extractions with oxalate, carbonate, 1 M KOH, 4 M KOH, chlorite, and 4 M KOH post chlorite (PC), listed in order of increasing severity, followed by screening of the extracts with monoclonal antibodies to determine the presence of diverse non-cellulosic glycan epitopes. Glycan epitopes present in later extractions indicate greater integration into the cell wall matrix. Antibody binding is indicated using a black-red-yellow scheme, where black indicates no antibody binding and yellow indicates maximal binding (as indicated by the gradient scale on the right of the figure). The key for cell wall glycan types is given along the right side of the profiles. The amount of material recovered in each extraction is indicated by a bar chart at the top of the figure in units of mg material recovered/gram cell wall

To further characterize how pretreatment affected the cell walls in the natural variants, the raw and pretreated *Populus* lines were subjected to glycome profiling, with the results shown in Fig. 5. Glycome profiling allows the identification and comparison of the relative abundance of glycan epitopes originating from the most major non-cellulosic plant cell wall glycans recovered in a series of six chemical extractions of increasing severity used to treat the solid residues. The monoclonal antibodies (mAbs) used in glycome profiling were epitope specific, thereby enabling conclusions about which glycan structures were present in each extract. Further, glycome profiling revealed the relative tightness with which these glycan epitopes were integrated into the cell walls, based on where epitopes were found in the series of cell wall extractions [18].

The results of the glycome profiling analyses (Fig. 5) provide several important insights for the raw *Populus* lines. First, oxalate and carbonate extractions removed more pectic backbone and pectic arabinogalactan epitopes from raw SKWE 24-2 and raw BESC 876 than from BESC standard. Overall, the pectic backbone and pectic arabinogalactan epitopes were easier to remove from raw SKWE 24-2 than from raw BESC 876. Thus, glycome profiling showed that the cell walls of the two untreated, low recalcitrant natural variants were overall more loosely integrated than the walls of the raw BESC standard. In general, pretreatment resulted in the removal of a significant amount of the glycan epitopes from the walls, as noted by the significant reduction/absence of most non-cellulosic glycan epitopes (except xylan, in general, and xyloglucans in BESC standard poplar) as indicated by the significantly lower binding of the mAbs against them (Fig. 5). SKWE 24-2 and BESC 876 were notably different from BESC standard regarding how they were affected by hydrothermal pretreatment. Pretreatment of SKWE 24-2 and BESC 876 biomass resulted in the complete removal of the base-extractable xyloglucan epitopes, while pretreated BESC standard still retained some of these epitopes. This explains the large amount of degradation products quantified for the low recalcitrant lines as soluble xylan/xyloglucan is readily degraded at elevated temperatures in the presence of an acid catalyst [19, 20].

The results of glycome profiling suggest that complete removal of extractable xyloglucans is essential for high sugar release from hydrothermally pretreated *Populus* by fungal enzymes and *C. thermocellum* (Fig. 5). The glycome profiling data also suggest that the pretreatment severity could be reduced for SKWE 24-2 and BESC 876 in that nearly all cell wall-extractable non-cellulosic carbohydrates other than xylan were removed during pretreatment. This result has important implications in that reducing pretreatment severity would reduce both operating costs and sugar losses.

Glycome profiling showed SKWE 24-2 to contain the most loosely bound (easily extractable) non-cellulosic glycans, followed by BESC 876 and then BESC standard. Overall, this information suggests that one could accurately predict the relative recalcitrance of these lines in their untreated and pretreated states based on glycome profiling alone. However, this conclusion warrants further testing with a larger number and diversity of natural variants. In any case, it is still important to validate the glycome profiling data by Stage 2 sugar release data to affirm its accuracy as a basis for drawing conclusions about recalcitrance as we have done here.

## Cellulose and hemicellulose chain length and biological conversion

**Fig. 6:**
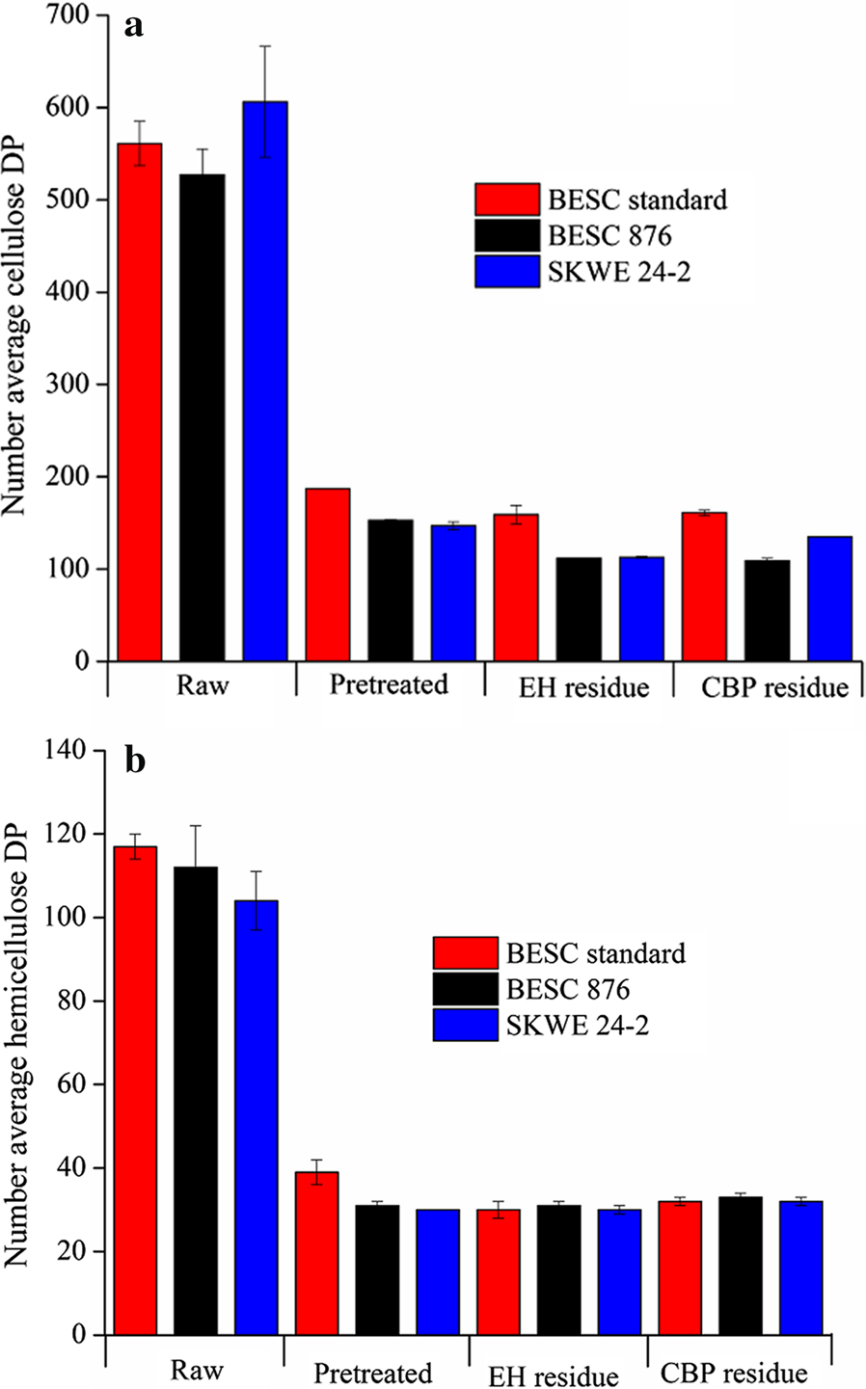
**a** Cellulose and **b** hemicellulose number-average degree of polymerization (DP_n_) for raw *Populus*, pretreated *Populus*, enzymatic hydrolysis (EH) residues, and *C. thermocellum* CBP (CBP) residues after 50% glucan plus xylan release. Enzymatic hydrolysis was carried out using 65 mg protein/g glucan in the pretreated biomass

Figure 6 shows cellulose and hemicellulose number-average degree of polymerization (DP_n_) for the raw, pretreated, and enzyme-hydrolyzed or *C. thermocellum*-digested *Populus* samples. Cellulose and hemicellulose DP_n_ were both greatly reduced after pretreatment with only minor to no reduction in the values after enzymatic hydrolysis at 65 mg protein/g glucan (EH) or deconstruction by *C. thermocellum* (CBP). The two low recalcitrant lines showed a greater reduction in cellulose and hemicellulose DP_n_ than BESC standard after pretreatment. These lines also had greater reductions in cellulose DP_n_, but not hemicellulose DP_n_, after enzyme or *C. thermocellum* hydrolysis than BESC standard. While *C. thermocellum* performed better than enzymes in Stage 2 digestion with more sugar released after 120 h, no distinction could be made between enzymatic and CBP residues despite terminating hydrolysis for both catalysts at 50% glucan and xylan release. Thus, cellulose and hemicellulose DP_n_ did not provide insights into how these two digestion processes differ. Cellulose and hemicellulose weight-average degree of polymerization (DP_w_) values were also evaluated; however, similar trends were observed for all samples and *Populus* lines as for DP_n_ (data not shown).

## Cellulose accessibility and its effect on glucan conversion

**Fig. 7:**
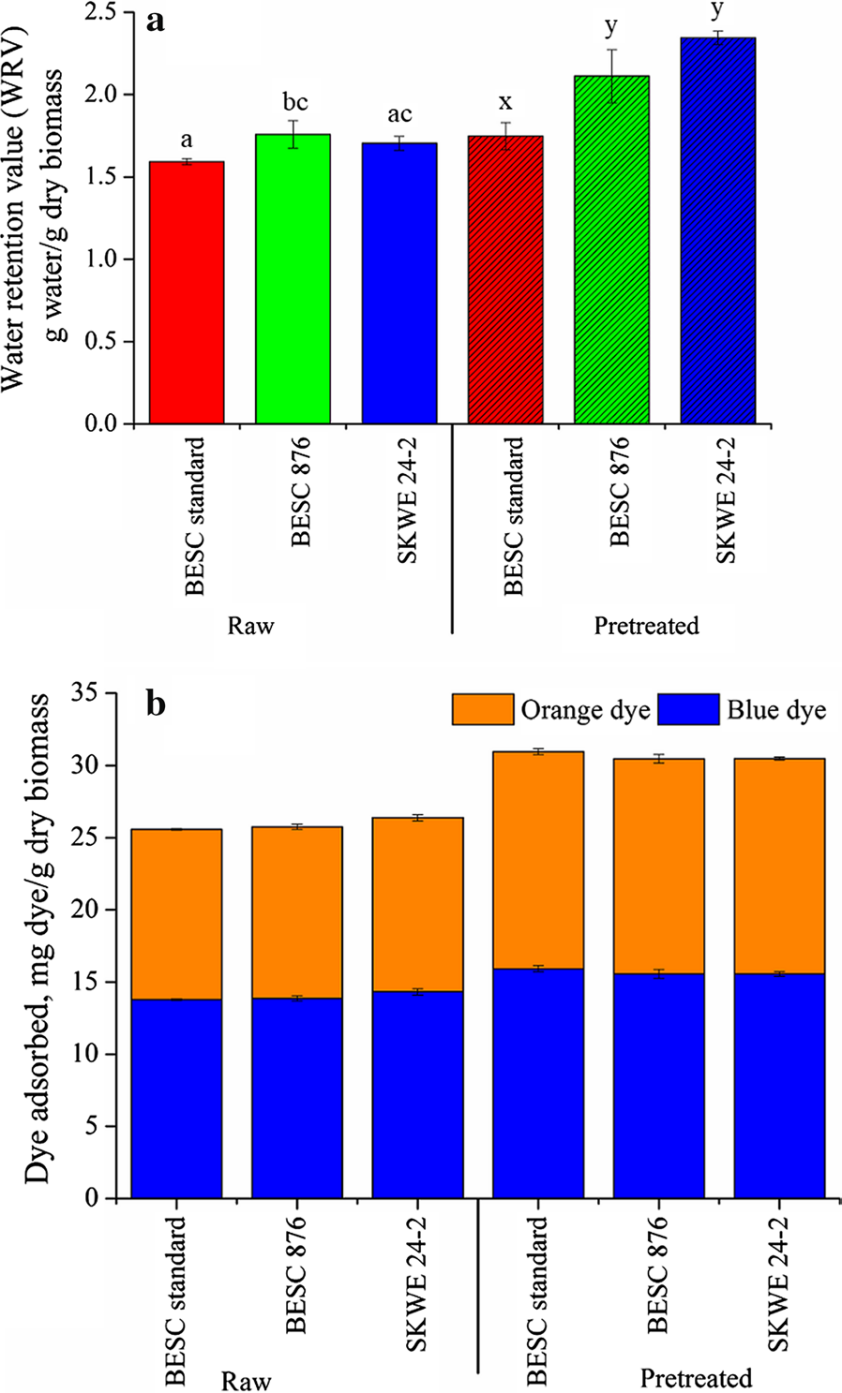
Effect of hydrothermal pretreatment on cellulose accessibility of natural variant Populus lines as measured by (**a**) water retention value and (**b**) dye adsorption via Simons’ staining method. Each sample was analyzed in triplicate. *p*-values for unpretreated and pretreated variants were 0.02669 and 0.00143, respectively. Columns with the same letter are not significantly different (*p* ≥ 0.05)

Water retention value (WRV) and Simons’ staining (SS) were applied to determine changes in cellulose accessibility that resulted from hydrothermal pretreatment, with the results shown in Fig. 7. Biomass recalcitrance has been strongly linked to limited cellulose accessibility to enzymes and/or microbes resulting in low digestibility [21, 22]. Water can form hydrogen bonds with accessible hydroxyl groups in cellulose. Thus, an increase in water retention can indicate an increase in cellulose surface area due to a larger number of sites being available for hydrogen bonding. Water retention in lignocellulosic biomass may, however, be affected by hydrogen bonding of water with the hemicellulose and pectin known to be present in the raw biomass. However, liquid hot water pretreatment at high temperatures, such as that used in this work, removes most of the minor glycan components of biomass including pectin (a high water-absorbing material), leaving a pretreated material containing mostly cellulose and lignin with small amounts of hemicellulose [23, 24]. This is evidenced by pretreated biomass composition data shown in Table 1, where these three major components of plant cell walls totaled nearly 99%, and by the glycome profiling data (Fig. 5). Thus, WRV after pretreatment can only be affected by cellulose and lignin and, to a lesser extent, by hemicellulose. It is also known that solids prepared by batch aqueous pretreatments have the major fraction of lignin as droplets or globules on the surface of cellulose, much of which is hydrophobic [25], thus leaving cellulose in pretreated biomass as the only (and decisive) component affecting retention of water. Other recent studies have also found strong linear correlations between WRV and enzymatic hydrolysis glucose yields [26, 27].

Modified Simons’ staining utilizes a high-molecular weight orange dye and a low-molecular weight blue dye of the direct dye class that bind to cellulose [28]. Cellulose surface area among samples can be inferred from total dye adsorption (orange + blue dye). On the other hand, an increase in the ratio of orange to blue dye in a sample can indicate the presence of larger pore sizes in the biomass. Both methods showed that pretreatment increased accessibility for all variants by removing much of the non-cellulosic glycans and some lignin. It can be observed from Fig. 7a that the WRVs of the three unpretreated variants were close to each other. Furthermore, dye adsorption data shown in Fig. 7b did not show any distinguishable differences among the three variants before or after pretreatment. However, WRVs of BESC 876 and SKWE 24-2 were significantly higher than that of BESC standard (Additional file 1: Table S1). This outcome may be due to the ability of much smaller water molecules to reach regions in biomass where the dyes cannot. On the other hand, it is possible that subtle differences in dye-binding capacity among variants may not have been detectable due to the dye concentration employed or ionic strength of the solution that are known to highly affect the adsorption of cellulosic dyes [29]. Moreover, differences in WRV or dyes’ adsorption could not have occurred due to variations in the constituents present in the biomasses as all three pretreated variants had highly similar chemical compositions. The WRVs of the low recalcitrant variants increased more after pretreatment than BESC standard, which is again consistent with the low recalcitrant variants being more digestible during Stage 2, as shown in Fig. 3.

## Lignin structure and composition and their relation to biological conversion

**Fig. 8:**
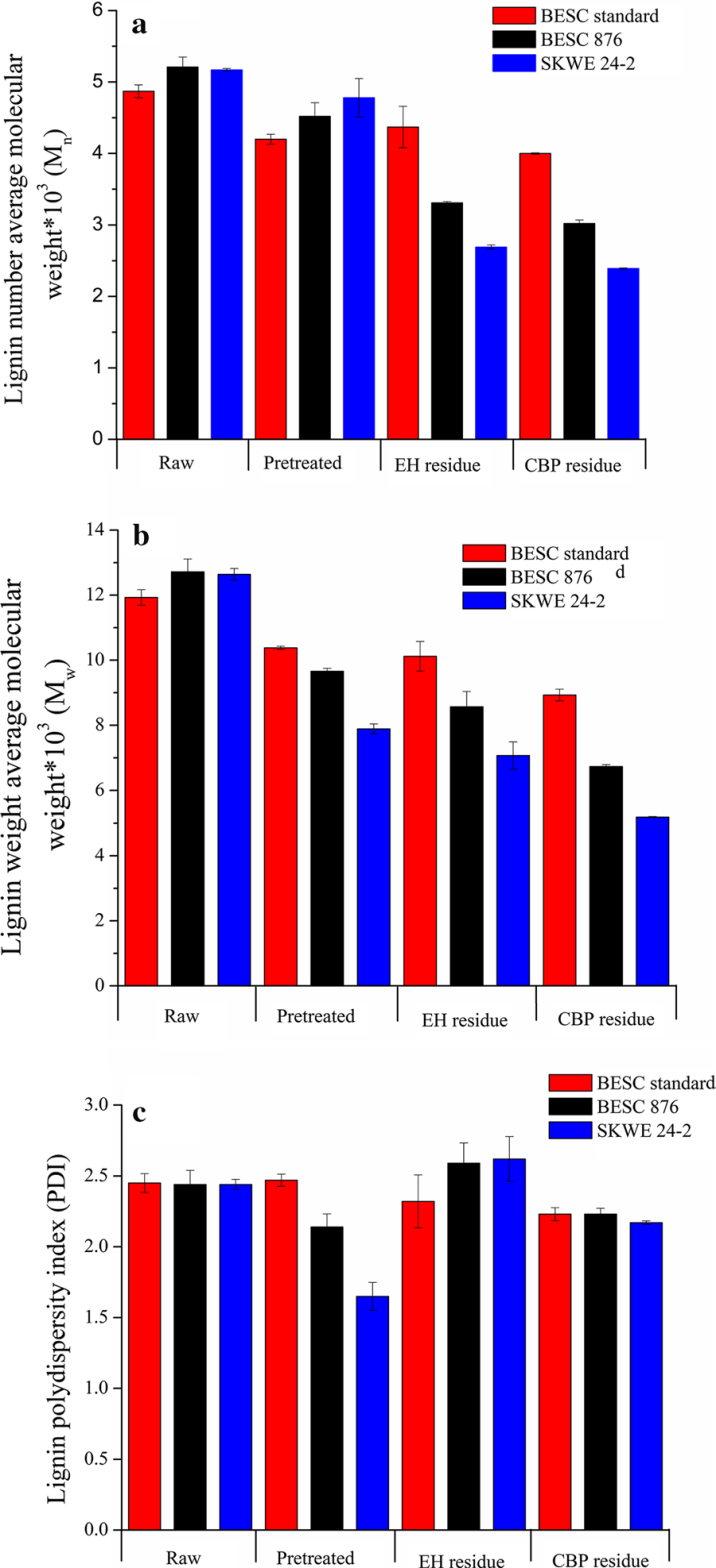
Lignin (**a**) number-average molecular weight, (**b**) weight-average molecular weight, and (**c**) polydispersity index for raw *Populus*, pretreated *Populus,* enzymatic hydrolysis (EH) residues, and *C. thermocellum* CBP (CBP) residues after 50% glucan plus xylan release. Enzymatic hydrolysis residues were prepared using a loading of 65 mg protein/g glucan in the pretreated biomass

Lignin number-average and weight-average molecular weight, *M*_n_ and *M*_w_, respectively, and polydispersity index (PDI), which is the ratio of *M*_w_ to *M*_n_, were determined for the raw biomass, pretreated solids, and biological residues, with the results presented in Fig. 8. Lignin *M*_n_ and *M*_w_ were reduced after hydrothermal pretreatment and further dropped after enzymatic and *C. thermocellum* digestion as shown in Fig. 8a and b, respectively. Greater reductions in lignin *M*_n_ and *M*_w_ occurred for the low recalcitrant lines compared to BESC standard. It was interesting that *M*_n_ and *M*_w_ converged for the pretreated, low recalcitrant *Populus* lines, thus resulting in a lower PDI, relative to their raw state, compared to BESC standard which had no change in lignin PDI after pretreatment. Furthermore, the greatest drop in PDI was observed for SKWE 24-2, followed by BESC 876 and lastly BESC standard, which had no change (see Fig. 8c). These results point to hydrothermal pretreatment forming a greater amount of low-molecular weight lignin for SKWE 24-2 and BESC 876 compared to BESC standard. A shift to lower molecular weight lignin after pretreatment of the low recalcitrant lines correlated positively with the results of hydrolysis in having greater glucan plus xylan release in Stage 2. Consequently, shorter lignin chains may be favorable for attaining effective hydrolysis by either free, fungal enzymes or *C. thermocellum* fermentations. Consistent with our findings, Meng et al. also reported that pretreated poplar natural variants that resulted in high sugar release in enzymatic hydrolysis had low-MW lignin [11]. However, the opposite was observed for unpretreated natural variants where natural variants that realized higher sugar release had higher molecular weight lignin [10].

Comparing lignin *M*_n_ and *M*_w_ for fungal cellulase and *C. thermocellum* residues shows that *C. thermocellum* produced consistently lower values across all lines. Thus, *C. thermocellum* was able to reduce lignin molecular weight better than enzymes. This may point to faster and/or more complete release of soluble hemicellulose fractions with bound lignin or of lignin alone by breaking cross-links within the biomass by *C. thermocellum*. Although neither the fungal enzyme cocktail nor *C. thermocellum* is known to directly digest lignin, one study has reported *C. thermocellum* to produce an enzyme capable of releasing coumaric acid, a cell wall component thought to have a role in cross-linking hemicellulose and lignin, from switchgrass and bagasse [30]. Identification of this enzymatic activity could explain why *C. thermocellum* reduced lignin molecular weight more than fungal enzymes.

**Fig. 9:**
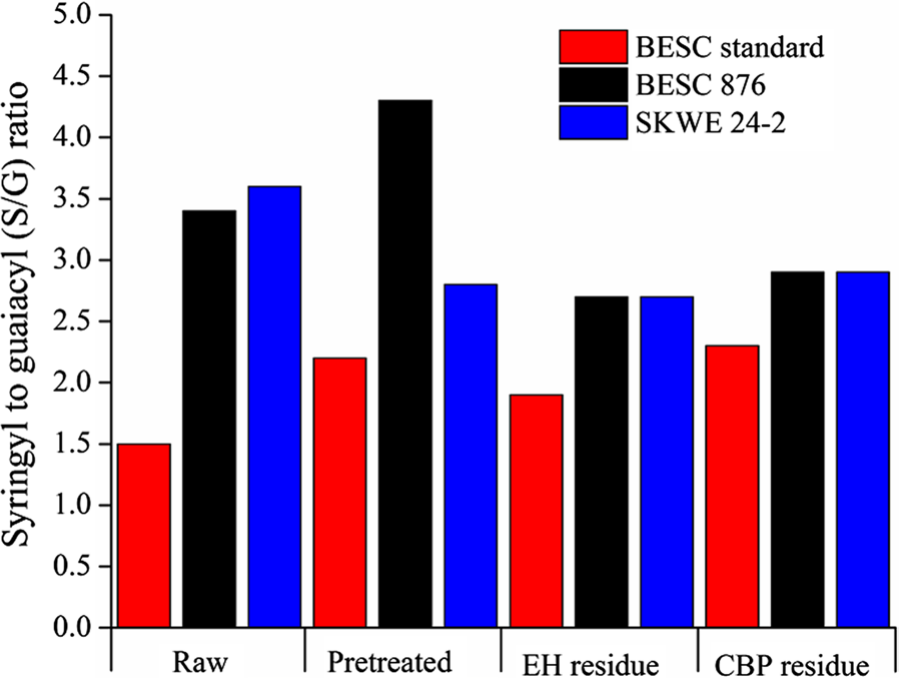
Lignin syringyl-to-guaiacyl ratios (S/G) were determined for raw, pretreated, and biological residues. Enzymatic hydrolysis (EH), carried out using a loading of 65 mg protein/g glucan of pretreated biomass, and *C. thermocellum* CBP (CBP) reactions were stopped at 50% glucan plus xylan release

**Fig. 10:**
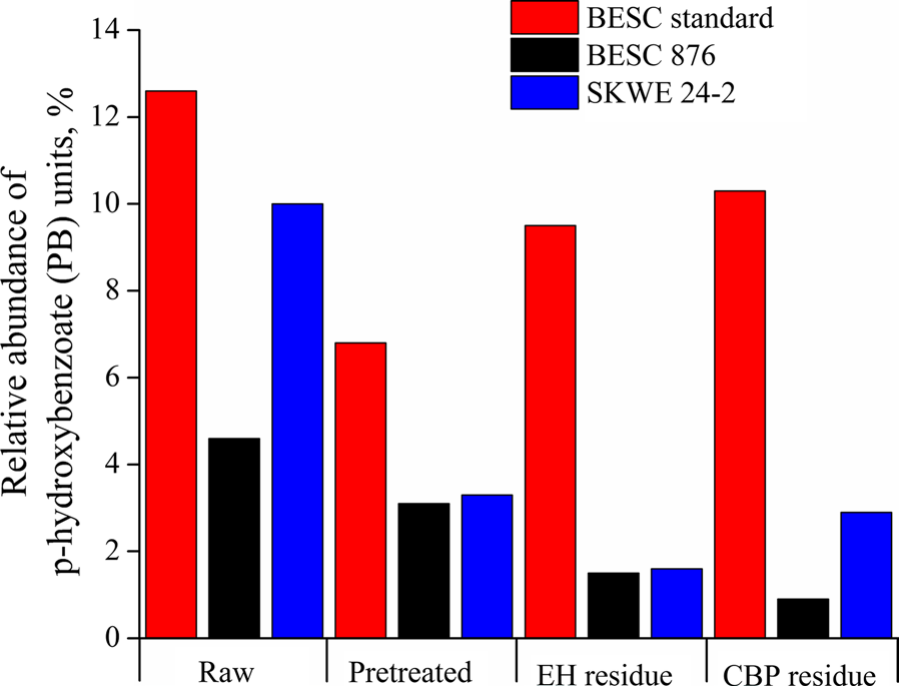
Relative abundance of *p*-hydroxybenzoate (PB) monolignol subunits was determined for raw, pretreated, and biological residues. Enzymatic hydrolysis (EH), carried out at a loading of 65 mg protein/g glucan of pretreated biomass, and *C. thermocellum* CBP (CBP) reactions were stopped at 50% glucan plus xylan release

Since many studies have demonstrated that lignin subunit ratios, in particular syringyl (S)-to-guaiacyl (G) ratios, are strongly correlated to sugar release [9], lignin functionalization was characterized by semi-quantification of the relative abundance of the monolignol subunits S, G, and *p*-hydroxybenzoate (PB) for raw, pretreated, and biological residues of the *Populus* lines. It can be observed from Fig. 9 that SKWE 24-2 and BESC 876 had higher lignin S/G ratios than BESC standard for raw, pretreated, and enzymatic hydrolysis/CBP residues. Although the lignin content in pretreated solids was not much different among three lines, the higher lignin S/G ratio in natural variants correlates well with the higher glucan digestion of these lines and is consistent with some studies in the literature [9]. However, Meng et al. showed an opposite trend between S/G ratio and glucose release [11]. Comparing biological catalysts, we see that across all three variants the lignin *S*/*G* ratios were higher for residues produced by *C. thermocellum* than for those left by enzymes. It can be observed from Fig. 10 that pretreatment reduced the amount of PB in all *Populus* lines, with the lowest PB quantities being observed in pretreated and biological residues of SKWE 24-2 and BESC 876. These results suggest that greater removal of the PB lignin subunit may be beneficial to increasing Stage 2 hydrolysis by either free fungal enzymes or *C. thermocellum*.

## Conclusions

In this study, we showed that screening for reduced recalcitrance in *Populus* can be an effective route to improve hydrolysis by both fungal enzymes and *C. thermocellum* after hydrothermal pretreatment. Additionally, *C. thermocellum* digestion proved to deconstruct hydrothermally pretreated solids substantially better than a commercial fungal cellulase cocktail, even when the latter was applied at extremely high loadings of 65 mg protein/g glucan in the pretreated biomass. Glycome profiling analyses suggested that the low recalcitrant natural variants have overall looser cell walls, especially with respect to pectic backbone and pectic arabinogalactan epitopes, than cell walls of BESC standard. Based on glycome profiling data, we hypothesize that complete removal of extractable xyloglucan from the pretreated *Populus* solids contributed to the reduced recalcitrance observed in biological digestion and may serve as a useful marker for identifying reduced recalcitrance. Lignin molecular weight characterization indicated that *C. thermocellum* may have the ability to fractionate lignin more efficiently than free, fungal enzymes, thereby reducing the obstruction of lignin to microbial action. Lignin characterization and WRV data revealed that PDI, WRV, and lignin *S*/*G* were useful markers for predicting the enhanced digestibility.

## Methods

### Experimental overview

**Fig. 11:**
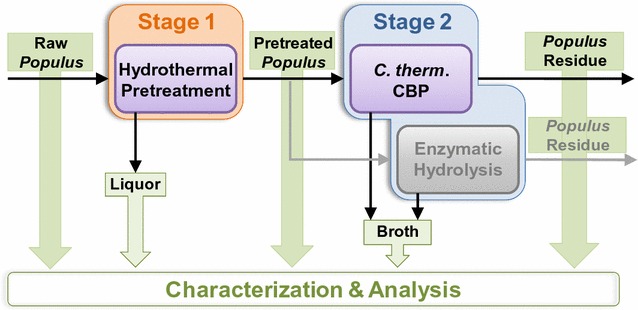
Overview of experimental characterization of deconstruction of three natural variant *Populus* lines. Raw *Populus* was processed by hydrothermal pretreatment (Stage 1) followed by biological deconstruction (Stage 2) with either *C. thermocellum* or fungal enzymes. The chemical compositions of all streams were measured along with various analyses of raw, pretreated, and biological residual solids

The experimental approach is summarized in Fig. 11. *Populus* was processed by hydrothermal pretreatment, Stage 1, followed by separation of the solids and liquid. The solids were washed with room-temperature deionized water before feeding them to *C. thermocellum* or fungal enzymes in Stage 2. The *Populus* residues noted in Fig. 11 were the solids that remained after Stage 2. Raw, pretreated, and residual *Populus* solids were characterized to determine the composition, molecular weights of cellulose, hemicellulose, and lignin, cellulose crystallinity, and the non-cellulosic glycan epitope compositions and extractabilities. The composition and mass of the Stage 1 (pretreatment) and Stage 2 (biological conversion) liquids were measured to complete material balances and determine sugar and metabolite concentrations.

### Substrates

BESC standard, BESC 876, and SKWE 24-2 *Populus* (*Populus trichocarpa*) were provided by the BioEnergy Science Center through Oak Ridge National Laboratory (ORNL, Oak Ridge, TN). BESC standard *Populus* was received, de-barked, and chipped with a moisture content of less than 10% (w/w). BESC 876 and SKWE 24-2 were received as logs freshly cut from trees grown in Clatskanie, OR. The logs were de-barked and chipped. The *Populus* biomasses were knife milled (Thomas-Wiley Laboratory Mill, Model 4, Thomas Scientific, Swedesboro, NJ) to a particle size of less than 1 mm using a 1-mm-size screen. All material that passed through the screen was collected, mixed together, divided into 1-gallon-size bags, and stored at – 20 °C. Microcrystalline cellulose powder, Avicel^®^ PH-101, was purchased from Sigma-Aldrich (St. Louis, MO) and stored at room temperature.

### Pretreatment

Biomass was soaked for a minimum of approximately 4 h prior to the reaction in deionized water. Hydrothermal pretreatments were performed at 5% (w/w) solid loading with a total weight of 750–800 g in a 1-L Hastelloy reactor (Parr Instrument Company, Moline, IL) equipped with a pressure gauge, thermocouple (Type K, Omega Engineering, Inc., Stamford, Connecticut), impeller, and electric motor (Pacific Scientific Automation Technology Group (Kollmorgen), Radford, VA). The reactor was heated to the desired temperature by lowering it via a chain hoist into a fluidized sand bath (Model SBL-2D, Techne, Princeton, NJ) maintained at 350–375 °C depending on the final reaction temperature [31]. The contents were mixed at 180 rpm. Heat-up times were recorded as the time for the reactor to rise from ambient temperature to within 2 °C of the target temperature, the error of the thermocouple. The reaction temperature was maintained by raising and lowering the reactor near the surface of the sand bath. The reaction was stopped by transferring the reactor to a room-temperature water bath with the cool-down time being the time for the reactor contents to cool from the target temperature to 80 °C. The pretreated solids and the resulting liquor were separated by vacuum filtration. Pretreatment liquor was collected and stored at − 20 °C. Filtered solids were collected, weighed, and stored at − 20 °C to prevent microbial degradation and subsequent compositional changes over time. Moisture content was determined by oven drying for determining pretreatment solid yield [32].

### Enzymatic hydrolysis

Enzymatic hydrolysis reactions were carried out at a pretreated solid loading of 5 g/L glucan in 125-mL flasks with a working volume of 50 mL at 50 °C and 150 rpm for up to 7 days in Multitron shakers (Model AJ125; Infors-HT, Laurel, MD, USA) according to the National Renewable Energy Laboratory (NREL, Golden, CO) procedure “Enzymatic Saccharification of Lignocellulosic Biomass” [33]. We have observed repeatedly with various biomass species that autoclaving alone does not have any major impact on sugar release in either CBP or enzymatic hydrolysis; thus, unlike CBP, where the biomass needs to be sterilized to control contamination, the pretreated solids used for enzymatic hydrolysis were not autoclaved. Instead, sodium azide at 0.2 g/L was used to prevent microbial growth. A 50 mM of sodium citrate buffer was added to maintain the reaction pH at 5.0 ± 0.1. Avicel^®^ PH101 was used as a substrate control to benchmark enzyme activity. Enzyme blanks without substrate were incubated with samples to determine the amount of sugar, if any, that was present in the enzyme solution. Fungal cellulase cocktail Accellerase^®^ 1500 (DuPont Industrial Biosciences, Palo Alto, CA; protein concentration ~ 86 mg/mL) was used at various loadings on a basis of mg protein/g glucan in the raw biomass [34]. Cellulase protein concentration was determined using a Pierce™ BCA protein assay kit with bovine serum albumin as a standard (ThermoFisher Scientific, Pittsburgh, PA).

### Anaerobic digestion/consolidated bioprocessing

*Clostridium thermocellum* strain DSM 1313 was obtained from Dr. Lee Lynd’s laboratory at Dartmouth College (Hanover, NH). Seed inoculum was prepared from a single batch of a single-colony isolate of exponential phase *C. thermocellum* cultured on MTC medium [35] and Avicel^®^ PH101 at 60 °C and 180 rpm. The chemicals used for preparing the media were obtained from Sigma-Aldrich (St. Louise, MO) or Fisher Scientific (Pittsburgh, PA). The seed inoculum batch was divided into 4-mL aliquots and stored at − 80 °C. Freezer stocks were cultured on Avicel^®^ PH101 at a concentration of 5 g glucan/L using MTC medium (less trace elements and yeast extract) with an inoculum concentration of 2 v/v%. Transferred freezer stock cultures were used to inoculate experimental samples of either biomass or cellulose loaded at 5 g glucan/L with a working volume of 50 mL.

All cultures and media were prepared in serum bottles, plugged with a butyl rubber stopper (Chemglass Life Sciences, Vineland, NJ), and sealed with an aluminum crimp. To make the contents of the bottle anaerobic, the headspace was flushed with nitrogen gas and then evacuated using a compressor (model ABF63 4B 7RQ, ATB, Vienna, Austria) for 45 s each. The flush/evacuation cycle was repeated 15 times. Biomass and substrates were sterilized by autoclaving and media were sterilized by autoclaving or filter sterilization (0.22-μm filter, Millipore, Billerica, MA) for heat-sensitive compounds. Bottle fermentation pH was maintained at pH 7.0 using MOPS buffer. Samples were taken at 12- or 24-h intervals for 7 days.

CBP residues were collected for structural polysaccharide and lignin quantification by centrifuging the entire reactor contents at 2800 rpm, removing the broth for HPLC analysis, and washing the solids 3 times with 50 mL (a total of 150 mL) of deionized water, vortexing the solids and water between washings. Residual solids were dried and weighed prior to structural polysaccharide and lignin quantification to determine the total weight loss.

### Structural sugar and lignin quantification

Solid samples from raw, pretreated, and CBP biomass solid residues were analyzed for structural sugar and lignin contents according to the NREL procedure “Determination of Structural Carbohydrates and Lignin in Biomass” [36]. Wheat straw (RM 8494) or Eastern Cottonwood Whole Biomass Feedstock (RM 8492) from the National Institute of Standards and Technology (NIST, Gaithersburg, MD) was run with each composition analysis as a standard reference material. If less than 300 mg of solids remained after fermentation, the procedure was scaled down according to the available sample weight [37].

Liquid samples from the pretreatment liquor, enzymatic hydrolysis reaction solution, and CBP broth were analyzed for soluble sugar monomers and oligomers by HPLC. The Waters HPLC, separations module e2695 with refractive index detector 2414 (Milford, MA) was operated with a 50 mM sulfuric acid solution eluent and an Aminex HPX-87H column (Bio-Rad, Hercules, CA) for separation of cellobiose, glucose, xylose, arabinose, formate, lactate, acetate, levulinic acid, ethanol, 5-HMF, and furfural. A minimum of two to five replicates were run for each analysis. To analyze sugar monomers, 30 μL of 10% (w/w) sulfuric acid solution was added to 1 mL enzymatic hydrolysis and CBP liquid samples to contain reactions, vortexed, and centrifuged to remove solids and cell debris prior to analysis. To quantify soluble oligomers, post-hydrolysis was performed as outlined in the NREL procedure, “Determination of Structural Carbohydrates and Lignin in Biomass” [36]. The sugar release calculations and Stage 1 material balance calculations were performed as described in detail elsewhere [16].

### Simons’ staining

Raw and pretreated biomasses from *Populus* natural variants were compared using a modified Simons’ staining method [38] carried out using Direct Orange 15 (CAS: 1325-35-5) and Direct Blue 1 (CAS 2610-05-1) which were generously donated by Pylam Products Company, Inc. (Tempe, Arizona). Direct Orange 15 was filtered through an EMD^®^ Millipore^®^ Amicon^®^ ultrafiltration apparatus using a 30,000 kDa cutoff polyethersulfone membrane (EMD^®^ Millipore^®^). The concentration of filtered dye was determined by drying three replicates of 1 mL filtered dye for 24 h and recording the initial and final weights. Direct Blue was used as-is. The filtered orange and blue dyes were mixed so that the final concentration of each dye in the mixture was 10 mg/mL. Serial dilutions were made from the stock solution of a mixture of dyes to obtain the calibration curve with absorbance in the range of 0.2–0.7 in accordance with Beer–Lambert–Bouguer’s law. Undried substrate (100 mg) was added to a 20-mL serum vial followed by 1 mL of phosphate buffer (0.3 M, pH 6.8), 1 mL of 1% NaCl solution, and 1 mL of dye mixture. The final volume was brought to 10 mL using deionized Milli-Q water. The vials were capped and shaken at 200 rpm in an incubator (Multitron Infors^®^HT Biotech, Laurel, MD) for 24 h at 60 °C. The dye concentrations in solution after equilibrium were measured on a SpectraMax^®^ M2e UV/VisPlate Reader (Molecular Devices, Sunnyvale, CA) equipped with SoftMax^®^ Pro data acquisition software in a Costar^®^ UV 96-well plate at 410 and 600 nm. Absorbance of a water blank was taken into account for correction to the sample absorbance. Three replicates for each sample in a 96-well plate were kept for measurements. The dye adsorbed on substrate and maximum orange-to-blue dye adsorption ratios were calculated from the concentrations of the remaining dyes in solution at equilibrium.

### Water retention value

A modified version of TAPPI Useful Method UM 256 [39] was applied for determination of water retention value (WRV) for raw and hydrothermally pretreated biomass from the *Populus* natural variants. Determination of WRV was carried out in ultrafiltration devices (EMD Millipore^®^ Ultrafree-CL Product# UFC40SV25) with a volume of 2 mL and Durapore^®^ PVDF membrane with a pore size of 5 µm. Three replicate ultrafiltration tubes were kept for each biomass sample. Also, three replicates of Avicel^®^ PH-101 were kept for a comparison with biomass samples. First, the moisture content of samples was determined using a halogen moisture analyzer (HB43-S; Mettler Toledo, Columbus, OH). The ultrafiltration tubes were dried at 40 °C for 12 h and then the filter inserts were weighed. This was the empty tube weight (*W*1). Then, based on the moisture content, approximately 90 mg of biomass on a dry basis of never-dried sample was loaded into tared tube inserts. The filter inserts were then inserted in collection tubes of the ultrafiltration devices. Milli-Q water (2 mL) was then added to the filter inserts and water-saturated samples allowed to soak for 12 h at room temperature. The devices were then spun in a moving bucket centrifuge (Allegra X-15R, Beckman Coulter, Fullerton, CA) at exactly 900*g* for 30 min at 21 °C. The tube inserts were weighed after centrifugation. This was the wet weight (*W*2). The devices were then dried at 105 °C in a gravimetric oven (Model# 6520, Thermo Electron Corp. Marietta, OH) for 12 h. Dried devices were allowed to cool in a desiccator for 15 min and weighed. This was the dry weight (*W*3). Water retention value is defined as the ratio of the mass of water retained in the sample after centrifugation to the mass of dry sample after centrifugation.

$${\text{WRV}} = \frac{W2 - W1}{W3 - W1} - 1$$

### Lignin isolation

The extractive-free biomass was ball-milled using a Retsch PM 100 planetary mill at 580 rpm for 1 h 30 min. The ball-milled biomass was hydrolyzed using an enzyme mixture containing 0.1 mL of Cellic^®^CTec2 and 0.1 mL of Cellic^®^ HTec2 in 20 mL sodium acetate buffer solution (pH 5.0) at 50 °C for 48 h. After hydrolysis, lignin was extracted from the solid residues using 96% dioxane for 48 h. The extracted lignin in dioxane was recovered by rotary evaporation and freeze-dried for GPC and NMR analyses.

## 2D HSQC NMR analysis

About 30 mg of isolated lignin (see previous section) was dissolved in 0.5 mL of DMSO-*d*_6_ for NMR analysis. NMR spectra were acquired at 298 K using a Bruker Avance III 400 MHz console equipped with a 5-mm BBO probe. Two-dimensional ^1^H–^13^C heteronuclear single quantum coherence (HSQC) spectra were collected using a Bruker standard pulse sequence (‘hsquetgpsi2′). HSQC experiments were carried out with a 11 ppm spectral width in F2 (^1^H) dimension with 2048 data points, 190 ppm spectral width in F1 (^13^C) dimension with 256 data points, 0.5 s pulse delay, and a ^1^ *J*_CH_ coupling constant of 145 Hz. The number of scans of 128 or 320 was employed depending on the sample concentration. The central solvent peak (*δ*_C_ 39.5 ppm; *δ*_H_ 2.5 ppm) was used for chemical shift calibration. NMR data were processed using the TopSpin 2.1 (Bruker BioSpin) and MNova (MestreLab Research) software packages.

### Lignin molecular weight analysis

The weight-average molecular weight (*M*_w_) and number-average molecular weight (*M*_n_) of lignin were estimated using gel permeation chromatography (GPC) analysis. Before the analysis, the isolated lignin was acetylated with anhydrous pyridine and acetic anhydride mixture (1:1, v/v) at room temperature for 24 h. The mixture was then added to ethanol and dried with a rotary evaporator to remove the solvents before dissolving the dried residue in tetrahydrofuran (THF). GPC analysis was performed with the PSS SECcurity GPC 1200 system (PSS, Amberst, MA, USA) with four Waters Styragel columns (HR1, HR2, HR4, and HR5; Waters Corporation, Milford, MA, USA) and Agilent refractive index (RI) and ultraviolet (UV) detectors (Agilent Technologies, Inc, Santa Clara, CA, USA). THF was used as the effluent and the flow rate was 1.0 mL/min. Polystyrene was used as the standard sample for establishing the calibration curve. The data were processed with the PSS WinGPC UniChrom software (Build 4815, version 8.2).

### Cellulose and hemicellulose molecular weight analysis by GPC

The extractive-free samples were delignified by peracetic acid with 5.0 g loading per g biomass [40]. The solution consistency was adjusted to 5% (w/w) with deionized (DI) water and the holopulping was conducted at room temperature for 24 h with magnetic stirring. The solid residue, designated as holocellulose, was washed extensively with DI water (Milli-Q water with resistivity 18.2 MΩ cm at 25 °C) and air dried at room temperature for 24 h. A portion of the air-dried holocellulose (100 mg) was consecutively extracted at 25 °C with 17.5% (w/v) NaOH solution (5.0 mL) for 2 h, followed by 8.75% (w/v) NaOH solution (10.0 mL) for an additional 2 h. The alkaline slurry was then filtered and rinsed with 5 mL of 1% (w/v) acetic acid leading to a liquid fraction and a solid residue. The solid residue, namely α-cellulose, was washed with an excess of DI water and air dried for the analysis of cellulose DP. The liquid fraction, rich in hemicellulose, was adjusted to pH 6–7 with anhydrous acetic acid. Hemicellulose was then precipitated by adding three volumes of 100% ethanol to the liquid fraction. Hemicellulose was then obtained by centrifugation at 8000 rpm (267π rad/s) for 5 min and freeze-dried for 24 h.

The weight-average molecular weight (*M*_w_) and number-average molecular weight (*M*_n_) of cellulose were measured by GPC after tricarbanilation. Briefly, the α-cellulose was derivatized with phenyl isocyanate in an anhydrous pyridine system prior to GPC analysis. Size-exclusion separation was performed on an Agilent 1200 HPLC system (Agilent Technologies, Inc, Santa Clara, CA) equipped with Waters Styragel columns (HR1, HR4, and HR5; Waters Corporation, Milford, MA). Number-average degree of polymerization (DP_n_) and weight-average degree of polymerization (DP_w_) of cellulose were obtained by dividing *M*_n_ and *M*_w_, respectively, by 519 g/mol, the molecular weight of the tricarbanilated cellulose repeating unit. The molecular weights of hemicellulose were measured by an Agilent 1200 series HPLC system equipped with three columns of Ultrahydrogel 120, 250, and 500 (Waters Inc.) linked in series. The freeze-dried hemicellulose samples were dissolved in 0.2 M sodium hydroxide/0.1 M sodium acetate (pH 11.8) mobile phase (~ 1.0 mg/mL) directly and filtered through a 0.45-µm filter before GPC analysis. Number-average degree of polymerization (DP_n_) and weight-average degree of polymerization (DP_w_) of hemicellulose were obtained by dividing *M*_n_ and *M*_w_, respectively, by 138 g/mol, the molecular weight of the xylose repeating unit.

$$M_{\text{n}} = \frac{{\mathop \sum \nolimits M_{i} *N_{i} }}{{\mathop \sum \nolimits N_{i} }}$$

$$M_{\text{w}} = \frac{{\mathop \sum \nolimits M_{i} *M_{i} *N_{i} }}{{\mathop \sum \nolimits M_{i} *N_{i} }}$$

$${\text{DP}}_{\text{n}} = \frac{{M_{\text{w}} }}{{M_{\text{n}} }}$$

$${\text{DP}}_{\text{n}} = \frac{{M_{\text{w}} }}{{M_{\text{n}} }},$$

where *M*_n_ and *M*_w_ are the number-average and weight-average molecular weights, respectively; DP_n_ and DP_w_ are the number-average and weight-average degrees of polymerization, respectively; *N*_*i*_ is the number of moles with the molar mass of *M*_*i*_; and *M*_0_ is the molecular mass of repeating unit (519 g/mol in the case of derivatized cellulose and 132 g/mol in the case of hemicellulose).

### Cellulose crystallinity analysis by CP-MAS

The isolated cellulose samples were stored in a sealed container to prevent moisture loss. The NMR samples were prepared by packing the moisturized cellulose into 4-mm cylindrical Zirconia MAS rotors. Cross-polarization magic angle spinning (CP/MAS) NMR analysis of cellulose was carried out on a Bruker Avance 400 spectrometer operating at a frequency of 100.59 MHz for ^13^C in a Bruker double-resonance MAS probe head at a spinning speed of 10 kHz. CP/MAS experiments utilized a 5-µs (90°) proton pulse, 1.5-ms contact pulse, 4 s recycle delay, and 4000 scans. The cellulose crystallinity index (CrI) was determined from the areas of the crystalline and amorphous C_4_ signals using the following formula:

$$CrI = \frac{{A_{86\text{ - }92 {\text{ppm}}}}}{{A_{86\text{ - }92 {\text{ppm}}} + A_{79\text{ - }86 {\text{ppm}}}}}.$$

### Glycome profiling

The non-cellulosic plant glycan-directed mAbs used in glycome profiling were obtained from laboratory stocks (CCRC, JIM, and MAC series) at the Complex Carbohydrate Research Center (available through CarboSource Services; http://www.carbosource.net) or from BioSupplies (Australia) (BG1, LAMP). In brief, glycome profiling involved the preparation of Alcohol-Insoluble Residues (AIR) from various biomass materials followed by sequential extraction of the AIR using increasingly harsh reagents as described earlier [18]. The cell wall extracts thus obtained were ELISA screened on an equal carbohydrate basis against a comprehensive suite of plant glycan-directed mAbs [41] using a robotic system (ThermoFisher Scientific, Pittsburgh, PA). The ELISA responses are represented as heatmaps. The gravimetric amounts of carbohydrate materials recovered in each extract are represented as bar graphs on top of the heatmaps.

### Statistical analysis

ANOVA was done using OriginPro v. 8.6 (OriginLab Corp., Northampton, MA) at an α level of 0.05 and post hoc analysis using Bonferroni method.

## Additional file

**Additional file 1: Table S1.** Statistical analysis of cellulose crystallinity, lignin molecular weight and PDI, and water retention value (WRV) for raw, pretreated, and biological residues of *Populus* natural variants.

## Abbreviations

mAbs: monoclonal antibodies
CBP: consolidated bioprocessing
WRV: water retention value
SS: Simons’ staining
DP: degree of polymerization
BESC: BioEnergy Science Center
