# Supplementary material for: Comparative evaluation of Populus variants total sugar release and structural features following pretreatment and digestion by two distinct biological systems
Source: Biotechnol Biofuels. 2017 Nov 30;10:292. doi: 10.1186/s13068-017-0975-x (PMC5718110; doi:10.1186/s13068-017-0975-x)
Supplement: Supplementary file 1 — Additional file 1: Table S1. Statistical analysis of cellulose crystallinity, lignin molecular weight and PDI, and water retention value (WRV) for raw, pretreated, and biological residues of Populus natural variants. [file 13068_2017_975_MOESM1_ESM.docx]

| **Table S1.** Statistical analysis of cellulose crystallinity, lignin molecular weight and PDI, and water retention value (WRV) for raw, pretreated, and biological residues of *Populus* natural variants | | | | | | | |
| --- | --- | --- | --- | --- | --- | --- | --- |
| Substrate  feature | Type | One-way ANOVA | | | Posthoc analysis* | | |
|  |  | F-statistic | p-value | Result | BESC Standard | BESC 876 | SKWE 24-2 |
| Cellulose CrI, % | Unpretreated biomass | 41.43 | 0.00653 | significant | a | a | b |
|  | Pretreated biomass | 1.35 | 0.38168 | not significant | - | - | - |
| Lignin M_w_ | Unpretreated biomass | 4.69 | 0.11939 | not significant | - | - | - |
| Lignin M_n_ | Unpretreated biomass | 7.37 | 0.0695 | not significant | - | - | - |
| Lignin PDI | Unpretreated biomass | 0.14 | 0.87421 | not significant | - | - | - |
| Lignin M_w_ | Pretreated biomass | 297.63 | 3.55E-04 | significant | a | b | c |
| Lignin M_n_ | Pretreated biomass | 4.45 | 0.12671 | not significant | - | - | - |
| Lignin PDI | Pretreated biomass | 106.17 | 0.00164 | significant | a | b | c |
| Lignin M_w_ | EH residue | 22.92 | 0.01523 | significant | a | ab | b |
| Lignin M_n_ | EH residue | 50.71 | 0.00487 | significant | a | b | b |
| Lignin PDI | EH residue | 4.98 | 0.11138 | not significant | - | - | - |
| Lignin M_w_ | CBP residue | 582.40 | 1.30E-04 | significant | a | b | c |
| Lignin M_n_ | CBP residue | 1462.74 | 3.28E-05 | significant | a | b | c |
| Lignin PDI | CBP residue | 3.78 | 0.15122 | not significant | - | - | - |
| WRV | Unpretreated biomass | 7.04 | 0.02669 | significant | a | b | ab |
| WRV | Pretreated biomass | 23.64 | 0.00143 | significant | a | b | b |

alpha level = 0.05; *poplar variant with same letter are not significantly different
